# Evaluation of a Natural Phytogenic Formulation as an Alternative to Pharmaceutical Zinc Oxide in the Diet of Weaned Piglets

**DOI:** 10.3390/ani13030431

**Published:** 2023-01-27

**Authors:** George Papadomichelakis, Irida Palamidi, Vasileios V. Paraskeuas, Elisavet Giamouri, Konstantinos C. Mountzouris

**Affiliations:** Laboratory of Nutritional Physiology and Feeding, Department of Animal Science, Agricultural University of Athens, 118 55 Athens, Greece

**Keywords:** piglets, zinc oxide, phytogenics, digestibility, gut ecology

## Abstract

**Simple Summary:**

Post-weaning diarrhea in piglets is caused by intestinal dysbiosis, characterized as an undesirable disruption of the physiological intestinal microbiota, which is associated with several stress factors, such as the isolation of piglets from sows, the adaptation to new environment and morphological changes in the intestine due to the transition from milk to solid feed intake. Pharmaceutical zinc oxide has been effectively used to alleviate these effects. However, since the dietary supplementation with pharmaceutical doses of zinc oxide (ZnO) in weaning pigs has been recently phased out in EU, this study aimed to determine the effect of a natural phytogenic formulation on growth performance, nutrient digestibility and faecal microbiota composition and metabolic activity. Dietary inclusion of the natural phytogenic formulation significantly increased piglet performance and nutrient digestibility and reduced the *E. coli* and *C. perfringens* counts as well as the molar ratios of branched chain volatile fatty acids in faeces. In summary, the results of this study indicate that the tested natural phytogenic formulation could be considered as an effective alternative to pharmaceutical doses of ZnO to alleviate the challenges commonly occurring after weaning.

**Abstract:**

A natural phytogenic formulation (NPF) was tested as an alternative to pharmaceutical zinc oxide (ZnO) in weaned piglets with respect to growth performance, apparent total tract digestibility and faecal microbiota composition and metabolic activity. Two dietary NPF levels (NPF: 1000 and 2000 mg/kg diet) were compared to a positive control (ZnO: 3000 mg ZnO/kg diet) and a negative control (CON: no added ZnO or NPF) using 84 weaned piglets from 29 d to 78 d (days of age). Feed conversion ratio was improved (*p* < 0.05) in ZnO and NPF piglets were compared to CON at 50 d. Dry matter, organic matter and crude protein (*p* < 0.05) digestibility was improved in NPF piglets compared to CON at 57 d. Compared to CON, NPF inclusion reduced *E. coli* (*p* < 0.05) and increased *C. leptum* subgroup (*p* < 0.01) at 57 d and 78 d, and reduced *C. perfringens* subgroup (*p* < 0.05; at 78 d). The ZnO reduced (*p* < 0.001) *E. coli* and *C. perfringens* subgroup (*p* < 0.01) compared to CON at 78 d. Moreover, ZnO and NPF reduced molar ratios of branched chain volatile fatty acids (*p* < 0.05) compared to CON, while NPF also increased butyric acid (*p* < 0.05) at 78 d. In conclusion, the NPF appeared to be a promising alternative to pharmaceutical doses of ZnO.

## 1. Introduction

Post-weaning diarrhea in piglets is caused by intestinal dysbiosis, characterized as an undesirable disruption of the physiological intestinal microbiota which is associated with several stress factors, such as the isolation of piglets from sows, the adaptation to new environments and morphological changes in the intestine due to the transition from milk to solid feed intake [1]. It is quite frequent, mainly during the first two weeks after weaning, and results in increased morbidity and mortality which have a serious economic impact on pig farming [2].

The post-weaning disorders have been treated for a long time using diets supplemented with antibiotics or high (pharmaceutical) doses of zinc oxide (ZnO) [3]. It has been shown that ZnO improves the intestinal barrier function, reduces pathogenic bacteria proliferation such as *E. coli* and *Salmonella* spp. and boosts the immune system when added to feed at doses ranging from 2000 to 3000 mg/kg [4,5]. However, the use of such high doses of ZnO has been linked to many adverse effects. The bioavailability and absorption of Zn from ZnO is relatively low (50–70%) and as a result a significant percentage of administered Zn is excreted in faeces, thereby causing heavy soil pollution [6]. Moreover, the ZnO can have toxic effects on piglets as Zn can accumulate in excessive amounts in tissues, although this is likely to occur only at doses higher than 4000 mg/kg diet [7], and it can also increase the resistance of *E. coli* and *Salmonella* spp. [8]. For these reasons, dietary supplementation with pharmaceutical doses of ZnO in weaning pigs has been phased out since June 2022 in Europe (Commission Implementing Decision of 26.6.2017, C (2017) 4, 529 Final), thereby urging us to investigate effective alternative feed additives.

Conversely, phytogenics contain multiple biologically active compounds that have been reported to have positive effects on animal growth and health due to their antibacterial, anti-inflammatory, and antioxidant properties [9,10,11,12]. Furthermore, phytogenics may stabilize the intestinal microbiota, reduce post-weaning incidences, optimize feed utilization and improve piglet health and growth [13,14,15,16,17,18]. Given the above, phytogenics could be considered as promising ZnO alternatives in weaning piglet diets [9,19,20]. However, while most of the relevant research has focused on purified phytogenic compounds, it is still not clear whether individual compounds or mixtures of phytogenic compounds could be more effective compared to the use of sole plants or mixtures of phytogenic plants [21,22]. In addition, limitations related to the phytogenic composition of raw materials, seasonal variations, the extraction and downstream processes, including storage effects, can introduce serious phytogenic variations that may affect phytogenic efficacy [23,24]. To our knowledge, there are no studies comparing natural phytogenic formulations comprising standardized mixtures of Mediterranean phytogenic plants directly to pharmaceutical doses of ZnO [25].

Thus, the objective of the present study was to assess the efficacy of a natural phytogenic formulation (NPF) based on olive polyphenols, alicin, apigenin and anethole as an alternative to pharmaceutical doses of ZnO in weaned piglets. The effects of two dietary inclusion levels of the NPF on growth performance, apparent total tract digestibility and faecal microbiota composition, and metabolic activity were compared to those of a negative control (no additives) and a positive (pharmaceutical dose of ZnO) control.

## 2. Materials and Methods

### 2.1. Animals and Diets

A total of 84 female [(Large White × Landrace) × Duroc] piglets, weaned at 28 days of age, were selected from a commercial farm. Upon arrival at the experimental facilities (29 days of age), they were weighed and allotted into 4 experimental treatments, with 7 replicates per treatment (3 pigs/replicate) balanced for body weight (average BW of 8.86 ± 0.525 kg; mean ± sd). Each replicate was kept in a pen (2.0 m × 0.9 m) with plastic slatted floor equipped with stainless feeders and nipple drinkers. During the nursing period on the farm, piglets had access to standard commercial pre-starter feed from the second week after birth. Piglets were allowed a 7-day pre-experimental period (from 29 to 35 days of age) which consisted of acclimatization to the new environment (3 days) and gradual transition to the experimental diets (4 days). No other feed additives or antibiotics were added to any of the experimental diets. All diets contained the minimum Zn content required for weaned piglets (100 mg/kg) from ZnO, according to the recommendations of NRC [26].

Experimental treatments consisted of 4 different diets: a negative control with no additives (CON); a positive control supplemented with 3000 mg ZnO/kg diet (ZnO); and two diets supplemented with 1000 and 2000 mg of a natural phytogenic formulation (NPF1 and NPF2, respectively) per kg diet. The natural phytogenic formulation (NPF) was a standardized mixture of Mediterranean plants specially formulated for piglets, having as main bioactive ingredients olive polyphenols, alicin, apigenin and anethole (NuPhoria; Nuevo SA, Sximatari, Greece), with a total bioactive content of 50 g/kg NPF.

Piglets had free access to feed and water throughout the experiment. Diets were formulated to meet or exceed the nutrient recommendations for weaned piglets weighing 7 to 11 kg [26] and were offered throughout the growing phase, following commercial practices. Diets had similar crude protein and metabolizable energy content; the ingredient and chemical composition are summarized in Table 1.

### 2.2. Experimental Procedures

The experiment was divided into 3 phases: from 29 to 50 (phase 1), from 51 to 64 (phase 2) and from 65 to 78 (phase 3) days of age. Phase 1 was longer because it included the pre-experimental period based on the consideration that valuable information regarding the effects of the experimental diets on piglets at early the post-weaning stage could be obtained. At the end of each phase, body weight and feed intake were recorded on a pen basis to calculate average daily gain (ADG), average daily feed intake (ADFI) and feed conversion ratio (FCR) for all treatment groups. A 2 h feed deprivation period was applied prior to weighing to avoid discrepancies in body weights due to differences in feed intake time. Piglets were observed daily for signs of diarrhea throughout the experiment.

One representative fresh faecal sample per pen (from all 3 piglets) was collected at three time points to determine fecal microbiota composition and metabolic activity (volatile fatty acid concentration): at the beginning of phase 1 (29 days of age;), at the mid point of phase 2 (57 days of age) and at the end of phase 3 (78 days of age). The three sampling time points are referred to as 29 d, 57 d, and 78 d. The samples were collected via massaging directly from the rectum of the piglets during the peak hours of defecation in sterile fecal container bags (Baglight, Intescience, Saint Nom la Bretêche—France). Samples were transported on ice to the laboratory and were stored at −80 °C until DNA extraction and real-time PCR analysis.

Two faecal collections from each pen were also carried out for the calculation of apparent total tract digestibility of dry matter (DM), organic matter (OM) and crude protein (CP). The first collection was performed daily from 55 to 57 days of age (mid phase 2) and the second from 76 to 78 days of age (end of phase 3). The two sampling time points are referred to as 57 d and 78 d. Prior to faecal sampling, animals were allowed a 7-day period during which they were fed their respective diets (C, ZnO, NPF1 and NPF2) containing 3 g titanium dioxide/kg feed as an indigestible marker. A representative sample of feed was taken from each feeder on the day prior to each faecal sampling. Faeces samples were collected in polyethylene bags from the floor of the pen and stored immediately at −80 °C until analysis. The pen floor was cleaned one day before the first day of collection and after each collection.

### 2.3. Chemical Analyses

For the calculation of apparent total tract digestibility, faecal samples were weighed and dried in a forced air oven (UF 750 plus, Memmert GmbH, Schwabach, Germany) at 60 °C for 72 h. Prior to analysis, feed and faecal samples were ground through a 1-mm screen in a laboratory mill (CT 293 Cyclotec, Foss, Hilleroed, Denmark). Routine procedures of AOAC [27] were used for ash (7.009) to calculate OM, DM (7.007), and EE (7.063) in feed and faecal samples. Crude fiber was determined using the filter bag system (ANKOM 220 Fiber Analyzer; ANKOM Technology, New York, NY, USA). The CP was determined as 6.25×Kjeldahl nitrogen, using a Kjeltec autoanalyzer unit (Foss, Hilleröd, Sweden). Titanium dioxide was determined in feed and faeces according to Myers et al. [28]. All analyses were performed in duplicate.

### 2.4. DNA Extraction and Quantitative Real-time PCR for Bacteria Enumeration

The fecal samples were used for DNA extraction using the suitable commercial kit (NucleoSpin™ DNA Stool, Macherey-Nagel GmbH & Co. KG, Düren, Germany). For each sample, the extracted DNA was eluted in a 80 μL elution buffer and the quality and quantity of the preparations were determined by spectrophotometry (Q3000, Quawell Technology, Inc., San Jose, CA, USA) and subsequently stored at −30 °C.

For the quantification of total bacteria (domain bacteria), *Lactobacillus* spp., *Escherichia coli, Bacteroides* spp., *Clostridium perfringens* subgroup (Clostridium cluster I), *Clostridium leptum* subgroup (*Clostridium cluster* IV) and *Clostridium coccoides* subgroup (*Clostridium cluster* XIVa), suitable primers were used which targeted the 16S rRNA gene (Table 2). 

Primer specificity was confirmed using the BLAST (NCBI) and PROBE MATCH programs ((Ribosomal Database Project II; Cole et al. [32]).

Real-time PCR was performed in microplates with the SaCycler-96 (Sacace Biotechnologies S.r.l., Como, Italy). Reactions were made at a 10 μL final volume and consisted of a 5 μL 2× FastGene IC Green universal mix (Nippon Genetics, Tokyo, Japan), as well as forward and reverse primers, each at a final concentration ranging from 300 to 450 nmol/L and with 1 μL of DNA template (20 ng sample DNA/reaction). The reactions were incubated at 95 °C for 5 s, at the primer-specific annealing temperature for 20 s, and at 72 °C for 33 s. This was followed by a melt curve analysis to determine the reaction specificity. Each sample was measured in duplicate. The samples results were expressed as log cells/g wet digesta contents.

Reference bacterial strains that were used to control the specificity of the primers and to construct standard curves are shown in Table 3. Each of the reference strains was cultured on selective nutrient broth under suitable conditions. Bacterial genomic DNA from each culture was extracted using a suitable commercial kit (NucleoSpin™ DNA Stool, Macherey-Nagel GmbH & Co. KG). For each sample, the extracted DNA was eluted in a 30 μL elution buffer and the quality and quantity of the preparations were determined by spectrophotometry (Q3000, Quawell Technology, Inc.) and stored at −30 °C.

For the quantification of bacterial species and groups, a quantification method similar to the one described by [33] was used. In more detail, an appropriate standard curve, using 10-fold serial dilutions of known concentrations of genomic DNA, was included on each 96-well plate. The number of genome copies, from each bacterial species in the initial purified DNA solution used to construct the standard curves, was calculated by assuming an average molecular mass of 660 Da for 1 bp of double-stranded DNA. The number of genome copies corresponds to an equal number of bacterial cells. Genome sizes for all bacteria species and groups used in this study are presented in Table 3.

### 2.5. Determination of Faecal Volatile Fatty Acids Concentration

For the determination of fecal volatile fatty acids (VFA) concentration, fecal samples were homogenized following a 10-fold dilution (i.e., 10% wt/vol) in sterile ice-cold phosphate-buffered saline (0.1 mol/L, pH 7.0). Homogenates were subsequently centrifuged at 12,000× *g* for 10 min at 4 °C and the resulting supernatants were stored at −80 °C until their analysis by capillary gas chromatography (GC). For the analysis, 85 μL of each supernatant was mixed with 10 μL 2-ethyl-butyrate (20 mM, internal standard) and 5 μL hydrochloric acid. Samples of 2 μL were injected into a gas chromatographer (Agilent 6890 GC System, Agilent Technologies, Santa Clara, CA, USA). The concentrations of VFAs were computed based on instrument calibration with a VFA standard mix (Supelco, Sigma Aldrich, St. Louis, MO, USA). Total VFA concentrations were expressed as mmol/g (wet weight) faeces and molar ratios of acetate, propionate, butyrate, and branched-chain VFAs (b-VFA; sum of iso-butyrate, iso-valerate, iso-caproic acid) and other VFAs (o-VFA; sum of valerate, caproic acid and heptanoic acid) were also calculated.

### 2.6. Statistical Analysis

Data were analyzed using the SPSS statistical package (version 17.0). Prior to analysis, data were tested for normality using the Kolmogorov–Smirnov test. Dependent variables that were not normally distributed were transformed according to a two-step approach which transforms the variable into a percentile rank and applies inverse-normal transformation to this rank to form a variable consisting of normally distributed z-scores [34]. Thereafter, planned contrasts were used to compare: (a) negative control with NPF-supplemented diets (C vs. (NPF1 and NPF2)) and (b) positive control with NPF-supplemented diets (ZnO vs. (NPF1 and NPF2)). Comparisons between C and ZnO diets were conducted by *t* test. The linear and quadratic effects of dietary NPF level were studied by polynomial contrasts using the data from C, NPF1 and NPF2 groups only. No significant diet by age interactions were observed for the parameters studied; hence, *p* values of interactions were not included in the tables. All data are presented as least squares (LSs) means ± root mean square error (RMSE). Pen was the experimental unit and statistical significance was set at *p* < 0.05. *p* Values, ranging from 0.05 to 0.10, were used to denote trends.

## 3. Results

### 3.1. Growth Performance and Digestibility

Piglet performance (feed intake, body weight gain, feed conversion ratio) for each experimental growth phase and the overall experimental period are presented in Table 4. The dietary addition of both ZnO and NPF significantly improved FCR (*p* = 0.041 and *p* = 0.019, respectively) compared to the control (CON) group from 29 to 50 days of age (Phase 1). No differences in growth performance between ZnO and NPF-fed piglets were detected. For the other two phases, there were no differences between diets; however, a trend for improved FCR (*p* = 0.057) and higher ADG (*p* = 0.061) was observed for NPF-fed piglets compared to the control (CON) group in Phase 2 and 3, respectively.

Polynomial contrasts results in Phase 1, showed that ADG increased and FCR decreased linearly (*P_linear_* = 0.040 and 0.014, respectively) with increasing dietary NPF content (Table 4). However, this linear effect was observed only for the period from 29 to 50 days of age of the piglets (Phase 1). From this age onwards (up to 78 days of age), no effects on performance of piglets were observed, with the exception of ADFI in Phase 2, which decreased linearly (*P_linear_* = 0.029) with increasing dietary NPF inclusion (Table 4) and resulted in a respective linear decreasing trend of FCR (*P_linear_* = 0.086).

In the present study, no differences in the coefficients of CATTD of DM, OM and CP between CON and ZnO-fed piglets were observed at 57 and 78 days of age (Table 5).

On the contrary, the CATTD of DM, OM and CP was greater (*p* = 0.035, *p* = 0.036 and *p* = 0.032) in the NPF treatments compared to CON fed piglets at 57 days of age. In addition, the CACTD of CP tended (*p* = 0.066) to be greater in the NPF when compared to the ZnO-fed piglets at 57 days of age. The CATTD of DM, OM and CP tended (*P*_linear_ = 0.061, *P*_linear_ = 0.065 and *P*_linear_ = 0.087, respectively) to increase linearly with increasing dietary NPF inclusion level at 57 days of age (Table 5). No differences between diets or linear effects of dietary NDF content were observed at 78 days of age with the exception of the CATTD of DM, which was greater in the NPF compared to ZnO (*p* = 0.038) fed piglets.

### 3.2. Faecal Microbiota Composition at the Designated Time Points (29, 57 and 78 d of Age)

There were no significant differences in the counts of total bacteria, *Bacteroides*, *Clostridium coccoides* subgroup, *Clostridium leptum* subgroup, *Clostridium perfringens* subgroup, *Lactobacillus* spp. and *Escherichia coli* between the experimental treatments at 29 days of age (Table 6).

At 57 days of piglet age, differences in the faecal *Clostridium leptum* subgroup and *Escherichia coli* were detected between the experimental treatments. The *Clostridium leptum* subgroup increased (*p* = 0.006) and the *Escherichia coli* decreased (*p* = 0.017) significantly in the faeces of NPF-fed piglets compared to CON piglets.

At 78 days of age, faecal *Clostridium leptum* subgroup, *Clostridium perfringens* subgroup and *Escherichia coli* differed between the treatments. Faecal *Clostridium leptum* subgroup increased (*p* = 0.004) and *Clostridium perfringens* subgroup, and *Escherichia coli* decreased (*p* = 0.038 and *p* = 0.002, respectively) in NPF-fed piglets compared to CON ones. The ZnO piglets also had a significantly smaller faecal *Clostridium perfringens* subgroup and lower *Escherichia coli* counts (*p* = 0.008 and *p* < 0.001, respectively) compared to the control. However, the *Escherichia coli* counts were significantly lower (*p* = 0.014) in ZnO when compared to the NPF piglets at the age of 78 days (Table 6).

Generally, dietary NPF inclusion level effects were seen for some of the changes noted in the faecal microbiota composition. In particular, the *Clostridium perfringens* subgroup counts decreased linearly (*P_linear_* = 0.034) with increasing dietary NPF at 78 days of age (Table 6). Furthermore, the faecal *Escherichia coli* counts decreased linearly with NPF inclusion level (*P_linear_* = 0.003) at 57 days of age and also decreased in a linear and quadratic pattern (*P_linear_* = 0.023 and *P_quadratic_* = 0.023, respectively) at 78 days of age with increasing NPF inclusion level.

Faecal total bacteria, *Bacteroides* spp., *Clostridium coccoides* subgroup and *Lactobacillus* spp. populations were not affected by the experimental treatments throughout the experiment.

### 3.3. Faecal Volatile Fatty Acid Concentration

At the beginning of the experiment (29 days of age), total VFA concentration, acetic acid, propionic acid, butyric acid, and the branched and other VFA molar ratio in the faeces did not differ between the experimental treatments (Table 7).

At 57 days of age, the acetic acid molar ratio was significantly lower (*p* = 0.050) in the faecal digesta of NPF in comparison with the ZnO-fed piglets. The other VFA (sum of valeric, caproic and heptanoic acids) molar ratio increased significantly (*p* = 0.003) in the NPF compared to the ZnO piglets and tended to be higher (*p* = 0.081) in the NPF compared to the CON piglets. In addition, the branched and the other VFA were also increased in a linear (*P_linear_* = 0.024) and quadratic pattern (*P_quadratic_* = 0.035), with increasing dietary NPF inclusion level.

At 78 days of age, the butyric acid molar ratio increased significantly (*p* = 0.026) in the faecal digesta of NPF in comparison with CON piglets and it also tended to increase (*p* = 0.076) in the ZnO when compared to CON piglets. In addition, the branched VFA molar ratio decreased significantly in ZnO- and NPF- (*p* = 0.031 and *p* = 0.037) fed piglets when compared to CON ones. The other VFA (valeric, caproic and heptanoic acids) molar ratio tended to be higher (*p* = 0.073) in the CON compared to NPF piglets. The effects of dietary NPF on faecal VFA concentration appeared to be dose-dependent again at 78 days of age. The molar ratio of butyric acid in faeces increased linearly (*P_linear_* = 0.002), whereas that of the branched VFA decreased linearly (*P_linear_* = 0.023) with increasing NPF inclusion level.

## 4. Discussion

Phytogenics may represent valuable tools available to the swine industry to alleviate weaning transition problems [35], with the main mechanisms for this being the modulation of the gut microbiota [20,36]. The present study aimed to assess the efficacy of a natural phytogenic formulation (NPF), based on a standardized blend of Mediterranean plants, as an alternative to pharmaceutical ZnO in weaned piglets. To achieve this objective, the effects of two dietary NPF contents on piglet performance, apparent total tract digestibility and faecal microbiota composition, and metabolic activity were studied at three (3) time points (e.g., 29, 57 and 78 d of piglet age). There were changes due to age but no significant diet interactions were found by age. Since the age effects on gut microbiota and other traits of the pigs have been thoroughly studied [37,38], this study focused primarily on the changes caused by the NPF inclusion compared to the negative (CON; no additives) and positive (ZnO) controls.

Regarding the impact of phytogenics on growth performance of pigs, literature results are variable. Our results showed differences in growth performance of the piglets receiving 1000 and 2000 mg NPF and 3000 mg ZnO/kg compared to the negative control (CON) treatment. The NPF and ZnO groups showed a significantly improved (lower) FCR than the CON group during Phase 1 post-weaning (from 29 to 50 d of age). Montoya et al. [12] studied the effects of phytogenics in *E. coli*-challenged piglets and reported a significantly improved FCR in ZnO-fed piglets only compared to control ones, while phytogenic fed piglets showed intermediate values. On the other hand, Kommera et al. [19] reported an FCR improvement in weaned piglets that were offered diets containing anise, citrus, and oregano oils. Similarly, Xun et al. [9] reported that a high dietary dose of curcumin significantly reduced the FCR compared to the non-treated group, but the final weight and ADG were not affected. Shi et al. [39] also noted that 300 mg curcumin/kg diet and the combination of phytogenics (curcumin and piperine), in addition to several other beneficial effects, significantly lowered the FCR compared to the non-treated group. In contrast, Namkung et al. [40] found a negative effect on FCR in piglets fed diets with cinnamon, thyme, and oregano oils during the first post-weaning week. This was attributed to lower ADG and ADFI due to the strong smell of the supplemented diet. The latter indicates that the level of supplementation and the composition of the essential oils need to additionally be considered carefully in terms of feed palatability.

The beneficial effect of NPF on FCR could be partially attributed to the improved dry matter, organic matter and crude protein digestibility observed in the NPF-fed piglets herein. Several works have reported an enhanced nutrient digestibility in response to supplemental phytogenic additives [41,42,43,44] owing to changes in the morphology of the intestine, particularly the increased villus-to-crypt ratio which enhances the absorption of nutrients [35,45].

In this study, NPF had an antimicrobial impact on the faecal microbiota composition by decreasing *E. coli* 57 days post-weaning and the *C. perfringens* subgroup and *E. coli* 78 days post-weaning. It is known that *E. coli* is a potential pathogen for piglets and, depending on its pathogenicity, can cause post-weaning diarrhea in pigs [1]. A reduction of *E. coli* concentration in faeces has been previous reported by the addition of phytogenics containing thymol and cinnamaldehyde in 35-day-old [35] and 56-day-old piglets [46]. *Clostridium* cluster I, which contains 17 subgroups, is separated from other *Clostridium* clusters (e.g., *Clostridium* cluster XIVa or *Clostridium* cluster IV) since it mainly consists of pathogenic or potential pathogenic bacteria, including *C. perfringens* [47,48]. Numerous opportunistic infections are caused by *C. perfringens*, both in human and animals [49]. Regarding piglets, *C. perfringens* has been strongly linked with enteric diseases in newborn piglets [50], whereas it has been shown that it can be persistent in the environment and the intestinal tract of growing or adult pigs [51]. With regards to the purported beneficial members of the gut microbiota, NPF addition increased the concentration of *Clostridium* cluster IV. This cluster contains numerous butyrate-producing and fibrolytic species (*C. leptum*, *C. sporosphaeroides*, *C. cellulosi*, and *Faecalibacterium prausnitzii*), whose metabolic activities have a significant effect on the hosts’ gut health and also provide the host with additional sources of nutrients [52,53]. Regarding ZnO, one of the best-known applications of high levels in pig rearing is the prevention of post-weaning diarrhea caused by *E. coli* [4]. However, the antimicrobial effect of ZnO is not specific and has diverse effects on gut microbiota such as reducing *Lactobacilaceae, Streptococcaceae* and *Coliforms* [4,20]. In this study, ZnO excreted its antimicrobial activity by reducing *E. coli* and *Clostridium* cluster I concentration at 78-day-old piglets, whereas it had no impact on the other members of microbiota examined at any time point, suggesting that ZnO protected the intestine from pathogenic growth and potential damage. Although not directly comparable, Kim et al. [54] reported that *Coliforms* and *Clostridium* spp. were reduced by the supplementation of 2500 mg ZnO/kg diet 2 weeks after weaning.

In the present study, evidence of phytogenic modulation of faecal microbial metabolism was shown, as significant changes in the VFA pattern were noted between NPF supplementation and the negative control (CON) at 78 days of age. Among VFAs, butyric acid has received particular attention for its beneficial effects on both cellular energy metabolism and gut homeostasis [55]. In addition, butyric acid has been shown to increase disease resistance in piglets via induced host defense peptides and histone deacetylase inhibition [56]. Therefore, NPF inclusion positively affected the metabolic activity of the microbiota by increasing fecal butyric acid molar ratio for 78-day-old piglets. This result can be correlated with the aforementioned NPF effect on *Clostridium leptum* subgroup, since the latter contains butyrate-producing bacteria [55].

The reduced branched–VFA molar ratios observed in this study, caused by dietary NPF and also ZnO additionl, could be considered as a beneficial trait for the piglet gut ecology. Concentrations of branched VFAs may be used as an indicator fo the quantity of protein fermentation [57]. Therefore, elevated levels of branched VFAs detected in faeces may indicate an excess of protein reaching distal parts of the intestine, pointing to non-efficient gut function [58]. Excess protein fermentation in the gut is not desirable since harmful metabolites are produced, (e.g., ammonia, or biogenic amines) which, in addition to burdening the environment, have also been linked to negative effects on the epithelium [59,60].

The effect of ZnO and NPF, differentiated for VFA pattern, on 57 d-old-piglets was observed. In particular, the acetic acid decreased, whereas other VFAs molar ratios were increased by NPF compared to ZnO. Acetic acid is produced by a variety of gut bacteria, such as *Lactobacillus* spp., *Bifidobacterium* spp., *Akkermansia muciniphila*, *Bacteroides* spp., *Prevotella* spp., *Ruminococcus* spp., and *Streptococcus* spp. via the Wood–Ljungdahl pathway and acetyl-CoA pathway [61]. However, no differences between NPF and ZnO were shown in microbial composition at the early stage of weaning (29–57 days). Perhaps a more detailed analysis at the genus level could shed further light towards elucidating a more detailed microbiota fingerprint.

It was noteworthy that the effects of dietary NPF on FCR, faecal microbiota composition and metabolic activity appeared to be dose-dependent; increasing NPF from 1000 to 2000 mg/kg diet resulted in a linear decrease in FCR at 57 days of age and also *E. coli* at 57 and 78 days and of *C. perfringens* subgroup faecal populations at 78 days post-weaning. In addition, faecal butyric acid increased and branched-chain fatty acids decreased linearly with increasing dietary NPF content. Based on these findings, further dose–response trials could be useful to further optimize dietary NPF inclusion levels.

## 5. Conclusions

In summary, the results of this study indicate that the tested natural phytogenic formulation could be considered an effective alternative to pharmaceutical doses of ZnO to alleviate the challenges commonly faced after weaning. The study of fecal material for microbiota composition and its metabolic activity has shown that phytogenic inclusion, and in particular the high dietary inclusion level (2000 mg/kg diet), significantly modulated the microbiota composition in a positive manner by increasing *C. leptum* subgroup levels, whilst reducing the *E. coli* and *C. perfringens* subgroup ones. At the same time, ZnO (3000 mg/kg) performed equally well with NPF at 57 days of age, while at 78 days of piglet age ZnO appeared more potent in reducing the *E. coli* levels compared to NPF. Furthermore, the VFA analysis, as a marker of microbiota activity, revealed that at 78 days of age, the phytogenic treatments increased butyric acid and reduced the branched–VFA molar ratios which is considered as a beneficial activity and complements to an extent the positive changes seen in the microbiota composition. Further work assessing piglet anti-inflammatory and anti-oxidant capacity at a molecular level is currently under way and it is expected to shed further light on phytogenic vs. ZnO modes of function in weaned piglets.

## Figures and Tables

**Table 1:** Ingredient and chemical composition of the experimental diets.

	Diet ^a^			
	ZnO	Control (C)	NPF1	NPF2
Ingredients (g/kg)				
Maize	594.0	600.0	598.0	597.0
Soybean meal (440 g CP/kg)	280.0	280.0	280.0	280.0
Soya protein concentrate (530 g CP/kg)	50.0	50.0	50.0	50.0
Soybean oil	35.0	32.0	33.0	33.0
Calcium carbonate	11.0	11.0	11.0	11.0
Monocalcium phosphate	11.0	11.0	11.0	11.0
Sodium chloride	6.2	6.2	6.2	6.2
L-Lysine 80%	4.0	4.0	4.0	4.0
DL-Methionine 99%	1.5	1.5	1.5	1.5
L-Threonine 99%	1.3	1.3	1.3	1.3
Vitamin premix ^b^	1.0	1.0	1.0	1.0
Mineral premix ^c^	2.0	2.0	2.0	2.0
Zinc oxide (ZnO)	3.0	-	-	-
Natural phytogenic formulation (NPF)	-	-	1.0	2.0
Analyzed chemical composition (g/kg DM)				
Dry matter (g/kg)	881.10	880.30	880.60	880.70
Ash	69.80	67.36	68.14	69.04
Crude protein	228.92	229.58	229.39	229.25
Ether extract	71.73	68.61	69.61	69.60
Crude fiber	35.86	36.01	36.00	35.88
Calculated chemical composition (g/kg DM) ^d^				
Digestible energy (MJ/kg)	16.65	16.64	16.65	16.63
Metabolizable energy (MJ/kg)	15.90	15.89	15.90	15.88
Calcium	8.84	8.77	8.79	8.81
Total phosphorus	7.00	7.02	7.02	7.01
Lysine	15.55	15.56	15.56	15.56
Methionine + cystine	8.97	9.09	8.97	8.97
Threonine	10.10	10.11	10.11	10.11
Tryptophane	2.50	2.50	2.50	2.50
SID ^e^ Lysine	14.19	14.20	14.19	14.19
SID ^e^ Methionine + cystine	8.29	8.29	8.29	8.29
SID ^e^ Threonine	8.85	8.86	8.86	8.86
SID ^e^ Tryptophane	2.27	2.27	2.27	2.27

^a^ ZnO, positive control with 3000 mg ZnO/kg feed; C, negative control with no added ZnO or phytogenic; NPF1, 1000 mg of natural phytogenic formulation (NPF)/kg diet; NPF2, 2000 mg of natural phytogenic formulation (NPF)/kg diet. ^b^ Vitamin premix (NUV 92001, Nuevo S.A., N. Artaki, Greece) provided per kg of diet: 15,000 IU vitamin A (retinyl acetate), 2000 IU vitamin D_3_ (cholecalciferol), 100 mg vitamin E (DL-α-tocopheryl acetate), 3.75 mg menadione (vitamin K_3_), 1.25 mg vitamin B_1_, 6 mg vitamin B_2_, 20 mg vitamin B_5_, 1.5 mg vitamin B_6_, 27.5 μg cyanocobalamin (B_12_), 32.5 mg nicotinic acid, 1 mg folic acid, 150 μg biotin. ^c^ Mineral premix (5502M, Nuevo S.A., N. Artaki, Greece) provided per kg of diet: 1.25 mg I, 0.3 mg Se, 150 mg Fe, 35 mg Mn, 50 mg Mg, 160 mg Cu and 100 mg Zn. ^d^ Digestible energy, macro-element and amino acid values for maize, soybean meal and whey proteins were adapted from tabulated data [26]. ^e^ Standardized ileal digestible.

**Table 2:** Primer sequences, targeting the 16S rRNA gene for the detection and quantitation of bacteria (according to Mountzouris et al. [29]).

Target Group or Organism	Sequence (5′-3′)	Annealing Temperature
All bacteria (domain bacteria)	F: ACTCCTACGGGAGGCAGCAG R: ATTACCGCGGCTGCTGG	60 °C
*Bacteroides* spp.	F: GAGAGGAAGGTCCCCCAC R: CGCTACTTGGCTGGTTCAG	58 °C
*Lactobacillus* spp.	F: GAGGCAGCAGTAGGGAATCTTC R: GGCCAGTTACTACCTCTATCCTTCTTC	60 °C
*Escherichia coli*	F: CATGCCGCGTGTATGAAGAA R: GGGTAACGTCAATGAGCAAAGG	60 °C
*C. perfringens* subgroup (*Clostridium* cluster I)	F: TACCHRAGGAGGAAGCCAC R: GTTCTTCCTAATCTCTACGCAT	56 °C
*C. leptum* subgroup (*Clostridium* cluster IV) *	F: GCACAAGCAGTGGAGT R: CTTCCTCCGTTTTGTCAA	52 °C
*C. coccoides* subgroup (*Clostridium* cluster XIVa) **	F: ACTCCTACGGGAGGCAGC R: CTTCTTAGTCAGGTACCGTCAT	60 °C

* Schwiertz et al. [30]; ** Matsuki et al. [31].

**Table 3:** Bacterial reference strains used for analytical process calibration purposes.

Reference Strains	Target Bacterial Group(s)	NCBI Reference Sequence	Genome Size, Mbp
*Escherichia coli* ATCC 25922	*Escherichia* sp. & domain bacteria	NZ_CP009072.1	5.13
*Bacteroides vulgatus* ATCC 8482	*Bacteroides* spp.	NC_009614.1	5.16
*Lactobacillus acidophilus* ATCC 314	*Lactobacillus* spp.	NC_006814.3	1.99
*C. perfringens* subgroup (*Clostridium* cluster I)	*C. perfringens* subgroup (*Clostridium* cluster I)	NC_008261.1	3.26
*Clostridium leptum* DSM 753	*C. leptum* subgroup (*Clostridium* cluster IV)	NZ_ABCB00000000.2	3.27
*Clostridium clostridioforme* DSM 933	*C. coccoides* subgroup (*Clostridium* cluster XIVa)	NZ_FOOJ00000000.1	5.47

**Table 4:** Effect of diet on body weight (BW, kg/piglet), average daily feed intake (ADFI, kg/piglet/day), average daily weight gain (ADG, kg/piglet/day) and feed conversion ratio (FCR, kg feed/kg gain) during the experiment.

	Diet ^a^				RMSE ^b^	Contrasts ^c^			Polynomial Contrasts ^d^	
	ZnO	C	NPF1	NPF2		C vs. ZnO	C vs. NPF	ZnO vs. NPF	*P_linear_*	*P_quadratic_*
Phase 1 (29–50 d of age)										
BW at 29 d	8.87	8.86	8.86	8.86	0.298	0.987	0.993	0.978	0.990	0.990
BW at 50 d	16.92	16.72	16.64	17.36	0.500	0.703	0.533	0.853	0.325	0.289
ADFI	0.564	0.571	0.564	0.598	0.025	0.777	0.631	0.421	0.409	0.304
ADG	0.383	0.369	0.380	0.412	0.016	0.389	0.066	0.371	0.040	0.269
FCR	1.47	1.552	1.491	1.449	0.038	0.041	0.019	0.993	0.014	0.823
Phase 2 (51–64 d of age)										
BW at 64 d	27.33	27.10	27.09	27.45	0.833	0.782	0.817	0.930	0.726	0.745
ADFI	1.193	1.202	1.170	1.137	0.036	0.790	0.131	0.222	0.029	0.626
ADG	0.744	0.738	0.755	0.733	0.027	0.824	0.799	0.998	0.979	0.392
FCR	1.603	1.638	1.555	1.553	0.048	0.481	0.057	0.251	0.086	0.560
Phase 3 (64–78 d of age)										
BW at 78 d	39.11	39.19	39.49	39.50	1.110	0.946	0.752	0.694	0.786	0.935
ADFI	1.536	1.477	1.573	1.537	0.045	0.208	0.064	0.626	0.189	0.189
ADG	0.848	0.839	0.896	0.883	0.029	0.757	0.061	0.102	0.321	0.321
FCR	1.804	1.787	1.773	1.705	0.049	0.742	0.269	0.144	0.384	0.384
Overall period (29–78 d)										
ADFI	1.019	1.032	1.029	1.025	0.030	0.649	0.824	0.757	0.782	0.938
ADG	0.613	0.614	0.626	0.638	0.016	0.951	0.218	0.172	0.803	0.803
FCR	1.629	1.671	1.650	1.609	0.035	0.245	0.194	0.981	0.492	0.492

^a^ ZnO, positive control with 3000 mg ZnO/kg feed; C, negative control with no added ZnO or phytogenic; NPF1, 1000 mg of natural phytogenic formulation (NPF)/kg diet; NPF2, 2000 mg of natural phytogenic formulation (NPF)/kg diet. ^b^ RMSE, root mean square error (*n =* 7 replicates/diet). ^c^ C vs. ZnO, difference between C and ZnO diets; C vs. NPF, contrast to compare the negative control with the NPF-supplemented diets (C vs. (NPF1 + NPF2)); ZnO vs. NPF, contrast to compare the positive control with the NPF-supplemented diets (ZnO vs. (NPF1 + NPF2)). ^d^ Polynomial contrasts used to test the linear and quadratic effect of dietary NPF using the C, NPF1 and NPF2 treatments, exclusively.

**Table 5:** Effect of diet on the coefficients of apparent total tract digestibility (CATTD) of dry matter (DM), organic matter (OM) and crude protein (CP) during the experiment.

	Diet ^a^				RMSE ^b^	Contrasts ^c^			Polynomial Contrasts ^d^	
	ZnO	C	NPF1	NPF2		C vs. ZnO	C vs. NPF	ZnO vs. NPF	*P_linear_*	*P_quadratic_*
57 d of age										
CATTD_DM_	0.832	0.828	0.841	0.838	0.0060	0.491	0.035	0.186	0.061	0.280
CATTD_OM_	0.849	0.845	0.858	0.855	0.0057	0.524	0.036	0.174	0.065	0.217
CATTD_CP_	0.77	0.767	0.790	0.786	0.0103	0.831	0.032	0.066	0.087	0.341
78 d of age										
CATTD_DM_	0.839	0.844	0.855	0.847	0.0063	0.390	0.234	0.038	0.422	0.170
CATTD_OM_	0.864	0.863	0.872	0.867	0.0061	0.893	0.237	0.299	0.394	0.314
CATTD_CP_	0.788	0.793	0.802	0.801	0.0106	0.633	0.379	0.163	0.434	0.710

^a^ ZnO, positive control with 3000 mg ZnO/kg feed; C, negative control with no added ZnO or phytogenic; NPF1, 1000 mg of natural phytogenic formulation (NPF)/kg diet; NPF2, 2000 mg of natural phytogenic formulation (NPF)/kg diet. ^b^ RMSE, root mean square error (*n =* 7 replicates/diet). ^c^ C vs. ZnO, difference between C and ZnO diets; C vs. NPF, contrast to compare the negative control with the NPF-supplemented diets (C vs. (NPF1 + NPF2)); ZnO vs. NPF, contrast to compare the positive control with the NPF-supplemented diets (ZnO vs. (NPF1 + NPF2)). ^d^ Polynomial contrasts used to test the linear and quadratic effect of dietary NPF using the C, NPF1 and NPF2 treatments, exclusively.

**Table 6:** Effect of diet on faecal microbiota composition (log cells/g wet faeces) of piglets at 29, 57 and 78 days of age.

	Diet ^a^				RMSE ^b^	Contrasts ^c^			Polynomial Contrasts ^d^	
	ZnO	C	NPF1	NPF2		C vs. ZnO	C vs. NPF	ZnO vs. NPF	*P_linear_*	*P_quadratic_*
29 d of age										
Total bacteria	9.72	9.66	9.65	9.72	0.054	0.241	0.583	0.413	0.395	0.336
*Bacteroides* spp.	9.04	8.91	8.98	9.08	0.105	0.226	0.207	0.891	0.122	0.603
*C. coccoides subgroup*	8.87	8.74	8.67	8.80	0.084	0.123	0.929	0.065	0.729	0.225
*C. leptum subgroup*	8.81	8.70	8.77	8.82	0.064	0.121	0.112	0.838	0.070	0.731
*C. perfringens subgroup*	7.91	7.69	7.53	7.72	0.143	0.142	0.577	0.029	0.931	0.210
*Lactobacillus* spp.	8.07	8.35	7.99	8.29	0.219	0.203	0.269	0.706	0.587	0.168
*Escherichia coli*	6.74	7.26	7.08	7.03	0.284	0.081	0.426	0.218	0.771	0.924
57 d of age										
Total bacteria	9.88	9.95	9.91	9.90	0.053	0.167	0.302	0.559	0.335	0.821
*Bacteroides* spp.	9.27	9.30	9.27	9.24	0.100	0.779	0.649	0.895	0.596	0.922
*C. coccoides subgroup*	9.36	9.26	9.49	9.46	0.125	0.428	0.060	0.309	0.100	0.302
*C. leptum subgroup*	9.19	9.08	9.27	9.26	0.069	0.141	0.006	0.208	0.302	0.387
*C. perfringens subgroup*	8.5	8.79	8.61	8.27	0.233	0.223	0.095	0.772	0.075	0.487
*Lactobacillus* spp.	8.87	8.72	8.76	8.65	0.196	0.443	0.940	0.319	0.617	0.617
*Escherichia coli*	5.53	6.08	5.72	5.22	0.276	0.056	0.017	0.801	0.003	0.332
78 d of age										
Total bacteria	9.94	10.03	10.01	10.06	0.074	0.206	0.935	0.132	0.764	0.532
*Bacteroides* spp.	9.27	9.31	9.27	9.39	0.124	0.767	0.840	0.591	0.412	0.412
*C. coccoides subgroup*	9.18	9.18	9.23	9.33	0.093	0.999	0.215	0.216	0.592	0.603
*C. leptum subgroup*	9.08	9.00	9.25	9.19	0.082	0.302	0.004	0.064	0.163	0.111
*C. perfringens subgroup*	8.34	8.95	8.76	8.34	0.207	0.008	0.038	0.271	0.034	0.325
*Lactobacillus* spp.	8.79	8.40	8.64	8.57	0.205	0.069	0.262	0.306	0.358	0.487
*Escherichia coli*	5.69	6.99	6.04	6.45	0.242	<0.001	0.002	0.014	0.023	0.023

^a^ ZnO, positive control with 3000 mg ZnO/kg feed; C, negative control with no added ZnO or phytogenics; NPF1, 1000 mg of natural phytogenic formulation (NPF)/kg diet; NPF2, 2000 mg of natural phytogenic formulation (NPF)/kg diet. ^b^ RMSE, root mean square error (*n =* 7 replicates/diet). ^c^ C vs. ZnO, difference between C and ZnO diets; C vs. NPF, contrast to compare the negative control with the NPF-supplemented diets (C vs. (NPF1 + NPF2)); ZnO vs. NPF, contrast to compare the positive control with the NPF-supplemented diets (ZnO vs. (NPF1 + NPF2)). ^d^ Polynomial contrasts used to test the linear and quadratic effect of dietary NPF using the C, NPF1 and NPF2 treatments, exclusively.

**Table 7:** Effect of diet on the total volatile fatty acid (VFA, mmol/kg of wet faeces) concentration and molar ratios of VFA (%) in the faecal digesta of piglets at 29, 57 and 78 days of age.

	Diet ^a^				RMSE ^b^	Contrasts ^c^			Polynomial Contrasts ^d^	
	ZnO	C	NPF1	NPF2		C vs. ZnO	C vs. NPF	ZnO vs. NPF	*P_linear_*	*P_quadratic_*
29 d of age										
Total VFA	60.5	88.0	58.8	67.6	14.08	0.062	0.108	0.759	0.281	0.281
Acetic acid	54.8	48.4	53.0	53.9	3.59	0.090	0.123	0.683	0.806	0.806
Propionic acid	17.4	17.8	16.4	17.7	1.80	0.817	0.621	0.819	0.449	0.449
Butyric acid	14.5	16.3	12.9	14.3	1.93	0.359	0.116	0.558	0.277	0.277
Branched VFA	10.2	9.8	8.9	10.5	1.17	0.724	0.910	0.607	0.256	0.256
Other VFA	3.8	6.1	4.4	3.9	1.35	0.100	0.113	0.763	0.859	0.859
57 d of age										
Total VFA	114.5	138.8	138.2	139.4	16.43	0.151	0.999	0.100	0.984	0.950
Acetic acid	48	45.9	45.0	45.1	1.69	0.225	0.539	0.050	0.610	0.753
Propionic acid	21.1	23.1	23.5	23.0	1.78	0.287	0.927	0.173	0.753	0.816
Butyric acid	24.5	22.9	24.0	21.7	2.30	0.502	0.995	0.436	0.745	0.363
Branched VFA	2.2	2.5	2.0	5.1	1.12	0.738	0.325	0.176	0.082	0.035
Other VFA	3.5	4.6	5.9	5.9	0.84	0.229	0.081	0.003	0.024	0.427
78 d of age										
Total VFA	122	156.8	129.3	144.9	16.42	0.053	0.200	0.300	0.397	0.217
Acetic acid	40.9	41.6	43.4	38.5	2.21	0.766	0.737	0.994	0.250	0.036
Propionic acid	21	21.0	20.2	22.1	1.37	0.998	0.880	0.882	0.536	0.197
Butyric acid	27.5	23.8	25.4	30.4	1.99	0.076	0.026	0.821	0.002	0.083
Branched VFA	5.2	7.5	6.2	5.0	1.00	0.031	0.037	0.664	0.023	0.720
Other VFA	6.4	7.5	5.8	6.4	0.90	0.228	0.073	0.659	0.109	0.199

^a^ ZnO, positive control with 3000 mg ZnO/kg feed; C, negative control with no added ZnO or phytogenics; NPF1, 1000 mg of natural phytogenic formulation (NPF)/kg diet; NPF2, 2000 mg of natural phytogenic formulation (NPF)/kg diet. ^b^ RMSE, root mean square error (*n =* 7 replicates/diet). ^c^ C vs. ZnO, difference between C and ZnO diets; C vs. NPF, contrast to compare the negative control with the NPF-supplemented diets (C vs. (NPF1 + NPF2)); ZnO vs. NPF, contrast to compare the positive control with the NPF-supplemented diets (ZnO vs. (NPF1 + NPF2)). ^d^ Polynomial contrasts used to test the linear and quadratic effect of dietary NPF using the C, NPF1 and NPF2 treatments, exclusively.

## Data Availability

The data analyzed during the current study are available from the corresponding author on reasonable request.

## Abbreviations

Zinc oxide (ZnO); natural phytogenic formulation (NPF); average daily gain (ADG); average daily feed intake (ADFI); feed conversion ratio (FCR); apparent total tract digestibility (CATTD); dry matter (DM); organic matter (OM); crude protein (CP); gas chromatography (GC); volatile fatty acids (VFA).
